# Crystal face dependent intrinsic wettability of metal oxide surfaces

**DOI:** 10.1093/nsr/nwaa166

**Published:** 2020-07-18

**Authors:** Zhongpeng Zhu, Zhenwei Yu, Frank F Yun, Deng Pan, Ye Tian, Lei Jiang, Xiaolin Wang

**Affiliations:** Key Laboratory of Bio-inspired Materials and Interfacial Science, Technical Institute of Physics and Chemistry, Chinese Academy of Sciences, Beijing 100190, China; Key Laboratory of Bio-inspired Smart Interfacial Science and Technology of Ministry of Education, School of Chemistry, Beihang University, Beijing 100191, China; Institute for Superconducting and Electronic Materials, Australian Institute for Innovative Materials, University of Wollongong, Wollongong, NSW 2500, Australia; Key Laboratory of Bio-inspired Smart Interfacial Science and Technology of Ministry of Education, School of Chemistry, Beihang University, Beijing 100191, China; Institute for Superconducting and Electronic Materials, Australian Institute for Innovative Materials, University of Wollongong, Wollongong, NSW 2500, Australia; Jinan Yian Biology Institute, Shandong Yian Biological Engineering Co. Ltd., Jinan 250100, China; Key Laboratory of Bio-inspired Materials and Interfacial Science, Technical Institute of Physics and Chemistry, Chinese Academy of Sciences, Beijing 100190, China; University of Chinese Academy of Sciences, Beijing 100049, China; Key Laboratory of Bio-inspired Materials and Interfacial Science, Technical Institute of Physics and Chemistry, Chinese Academy of Sciences, Beijing 100190, China; Key Laboratory of Bio-inspired Smart Interfacial Science and Technology of Ministry of Education, School of Chemistry, Beihang University, Beijing 100191, China; University of Chinese Academy of Sciences, Beijing 100049, China; Institute for Superconducting and Electronic Materials, Australian Institute for Innovative Materials, University of Wollongong, Wollongong, NSW 2500, Australia; ARC Centre of Excellence for Future Low-Energy Electronics Technologies (FLEET), University of Wollongong, North Wollongong, NSW 2522, Australia

**Keywords:** anisotropic wettability, α-Al_2_O_3_, interfacial water layer, surface energy, DFT simulation

## Abstract

Knowledge of intrinsic wettability at solid/liquid interfaces at the molecular level perspective is significant in understanding crucial progress in some fields, such as electrochemistry, molecular biology and earth science. It is generally believed that surface wettability is determined by the surface chemical component and surface topography. However, when taking molecular structures and interactions into consideration, many intriguing phenomena would enrich or even redress our understanding of surface wettability. From the perspective of interfacial water molecule structures, here, we discovered that the intrinsic wettability of crystal metal oxide is not only dependent on the chemical components but also critically dependent on the crystal faces. For example, the $( {1\bar{1}02} )$ crystal face of α-Al_2_O_3_ is intrinsically hydrophobic with a water contact angle near 90°, while another three crystal faces are intrinsically hydrophilic with water contact angles <65°. Based on surface energy analysis, it is found that the total surface energy, polar component and Lewis base portion of the hydrophobic crystal face are all smaller than the other three hydrophilic crystal faces indicating that they have different surface states. DFT simulation further revealed that the adsorbed interfacial water molecules on each crystal face hold various orientations. Herein, the third crucial factor for surface wettability from the perspective of the molecular level is presented, that is the orientations of adsorbed interfacial water molecules apart from the macro-level chemical component and surface topography. This study may serve as a source of inspiration for improving wetting theoretical models and designing controllable wettability at the molecular/atomic level.

## Teaser

The study of crystal face dependent wettability has led to the discovery of a hydrophobic crystal surface of α-Al_2_O_3_ single crystals.

## INTRODUCTION

The intrinsic wettability of the solid/liquid interface is a critical factor in fabrication of functional materials and devices [1–6]. From a general point of view, wettability difference is explicated as an average effect of surface chemical components and surface topography [7–11]. However, to further reveal the intrinsic wettability differences, molecular interactions at the liquid/solid interfaces should be addressed, with the possibility of uncovering new phenomena [12–14]. An intriguing example is that from the view of molecular interaction, the limitation of hydrophilic and hydrophobic surfaces is addressed at around 65° rather than 90° [15,16]. Detailed studies by surface force apparatus revealed that the long-range attraction force between two solids in water disappears for surfaces with water contact angle (CA) near 65°, compared with others around 90° [17,18]. Hence, it can be seen that analysis from the perspective of molecular interactions at liquid/solid interfaces may reveal more intrinsic properties concerning the wettability of solid surfaces [19–23].

Metal oxides are widely used in the modern industry, such as electrode materials for energy-storage devices, substrate materials for optoelectronic devices, and catalysts [24–26]. Detailed knowledge to understand and control the surface properties of metal oxides is a prerequisite for many industrial applications. Generally, clean metal oxide surfaces are treated as hydrophilic because of their high surface energy [27,28]. For example, it has been reported that clean alumina is hydrophilic because of the empty (3p) orbitals of the valance band at the solid surface, which acts as Lewis acid sites [29]. However, different arrangements of interfacial atoms can change the surface properties on each crystal face significantly [30,31]. Calculations using density functional theory (DFT) indicate that the surface energy of the Al_2_O_3_ crystal face can even be negative as a result of the dissociated water molecules on the surface [32]. So, the interfacial water molecules may have a critical role in the wettability of atomically flat solid surfaces. It is well-known that there are various kinds of crystal faces for Al_2_O_3_. Hence, to further reveal the intrinsic wettability at the molecular level, we chose four crystal faces of Al_2_O_3_, namely $( {11\bar{2}0} )$, $( {10\bar{1}0} )$, $( {0001} )$ and $( {1\bar{1}02} )$ as typical examples for further studies.

Herein, we report crystal face dependent wettability differences of α-Al_2_O_3_ resulting from the different orientations of adsorbed interfacial water molecules. It is found that the water CA of the $( {1\bar{1}02} )$ crystal face is around 90°, while three other crystal faces, namely $( {11\bar{2}0} )$, $( {10\bar{1}0} )$ and $( {0001} )$, are intrinsically hydrophilic, with water CAs <65°. Detailed analysis of these crystal faces found that the total surface energy, polar component and Lewis base portion of hydrophobic crystal faces are relatively lower than those of hydrophilic faces. DFT simulation revealed that the adsorbed interfacial water molecules are in a vertical position and dissociative state on the $( {1\bar{1}02} )$ crystal face. In this case, water molecules at the three-phase contact line are eager to form inner hydrogen bond networks rather than interfacial hydrogen bond networks, which results in a relatively hydrophobic state. Furthermore, different laser engraving patterns were fabricated on hydrophobic $( {1\bar{1}02} )$ crystal faces showing that the CA decreases gradually with increasing exposure of hydrophilic crystal faces. These findings demonstrated that orientations of adsorbed interfacial water molecules are the third crucial factor for surface wettability from the perspective of molecular level, apart from chemical component and surface topography at the macro-level, which are critically important in improving wetting theoretical models and wettability manipulation at the molecular/atomic level for single-atom catalysis.

## RESULTS AND DISCUSSION

**Figure 1.**
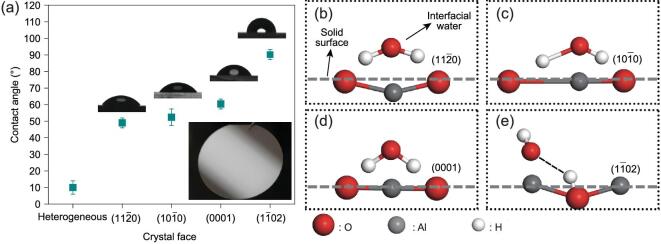
Macroscopic wettability differences of different α-Al_2_O_3_ crystal faces and schematic diagrams to illustrate the mechanism at the molecular level. (a) Polycrystalline alumina is highly hydrophilic, while α-Al_2_O_3_ crystal faces with $( {11\bar{2}0} )$, $( {10\bar{1}0} )$ and $( {0001} )$ orientations are hydrophilic, but the $( {1\bar{1}02} )$ crystal face is hydrophobic (inset shows the optical image of a mirror flat single crystal α-Al_2_O_3_ sample). Schematic diagrams show the composite structure of adsorbed interfacial water molecules and the exposed solid atoms. The orientations of water molecules will result in two typical statuses offering two lone pair electrons (b–d) or one hydrogen atom (e) at the topmost position.

The surface wettability of metal oxide is critically dependant on its crystal faces, which can be clearly seen in Fig. 1a on α-Al_2_O_3_. An X-ray diffraction technique was used to confirm that these α-Al_2_O_3_ are all phase pure (Supplementary Fig. 1). To maintain the original surface property of α-Al_2_O_3_ surfaces with clean crystal faces, these underwent a two-step cleaning procedure consisting of polishing and sonication. Before cleaning, water CAs of α-Al_2_O_3_ single crystal faces were high, as a result of adsorption of hydrocarbons and particles (Supplementary Fig. 2). This effect has been shown previously for a variety of rare-earth oxide and metal oxide materials, including Holmia, Ceria, zirconium and titanium dioxide [29]. As shown in Fig. 1a, after cleaning, the CAs of $( {11\bar{2}0} )$, $( {10\bar{1}0} )$ and $( {0001} )$ crystal faces decreased to 50.0 ± 3.1°, 54.3 ± 4.7° and 60.4 ± 3.0°, respectively. Surprisingly, the $( {1\bar{1}02} )$ surface retained a relatively high CA of 90.2 ± 2.5°. As these surfaces are mirror flat (optical inset image of Fig. 1a) with similar chemical composition, the results indicated that the wetting behaviors of α-Al_2_O_3_ single crystal faces are critically dependent on the crystal faces. It is suggested that these wettability differences may result from the composite structure of adsorbed water interfacial molecules and the arrangement of solid atoms at the surface. As shown in Fig. 1b–e, the adsorbed interfacial water molecules lying on the solid surface can offer four hydrogen bond interaction sites, namely two hydrogens and two lone pair electrons from the oxygen atom to interact with the bulk water molecules of water droplets. Based on the orientation of adsorbed interfacial water molecules, there are two typical situations. One is the oxygen atom at the top offering two lone pair electrons. In this case, water will form more hydrogen bonds with the solid surfaces to form interfacial hydrogen bond networks, resulting in hydrophilic behaviors (Fig. 1b–d). The other is a hydrogen atom at the top. In this case, water molecules will form more inner hydrogen bond networks within the liquid droplets rather than strong interactions with the adsorbed interfacial water molecules at the solid surface, which results in hydrophobic behaviors (Fig. 1e). Therefore, it is inferred that the wettability changes of different α-Al_2_O_3_ crystal faces may result from the orientation of adsorbed interfacial water molecules, i.e. with a decrease of interaction sites between free water molecules of water droplets and adsorbed interfacial water molecules at the solid surfaces, the crystal faces of α-Al_2_O_3_ become more and more hydrophobic.

**Figure 2.**
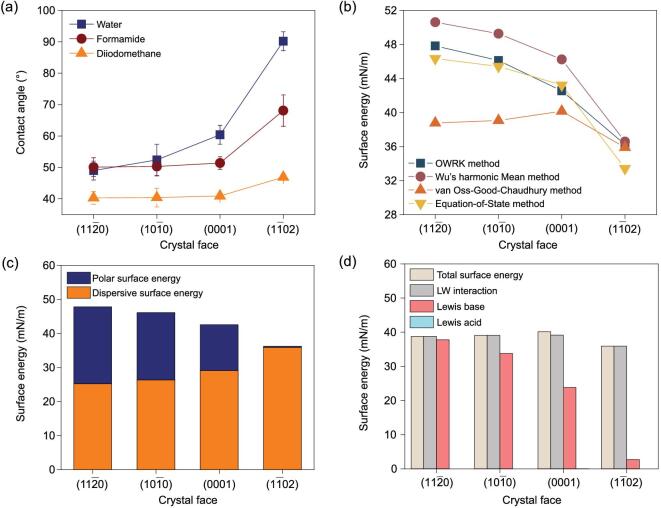
Detailed surface energy analysis based on various calculation methods. (a) CAs of three probe liquids were measured, revealing that the $( {1\bar{1}02} )$ crystal face showed the largest CA compared with the other three. (b) Calculation based on CA measurements with four different methods was applied to estimate the total surface energy. (c) The polar and dispersive components of each crystal surface were analyzed by the OWRK method. (d) Lewis acid/base portions of each crystal surface were analyzed by the van Oss-Good-Chaudhury method.

Surface energy analysis was applied to study the wettability difference of these α-Al_2_O_3_ crystal faces with CA measurements of three probe liquids and further calculation to estimate the total surface energy, polar/dispersive components and Lewis acid/base portions. As shown in Fig. 2a, CAs of the $( {11\bar{2}0} )$, $( {10\bar{1}0} )$ and $( {0001} )$ crystal faces are similar for each kind of liquid, while the CA of the $( {1\bar{1}02} )$ crystal face remains relatively high. To further reveal the detailed difference concerning the total surface energy, four different methods, the Equation-of-State method, Owens-Wandt-Rabel-Kaelble (OWRK), van Oss-Good-Chaudhury method and Wu's harmonic Mean method, were applied (Supplementary Tables 1–4). Results show that surface energy tendencies of $( {11\bar{2}0} )$, $( {10\bar{1}0} )$ and $( {0001} )$ crystal faces are similar in each method, but, in contrast, the $( {1\bar{1}02} )$ crystal face has the lowest surface energy (Fig. 2b). Furthermore, surface polar/dispersive components were analyzed by the OWRK method (Fig. 2c). The total surface energy is indicated by the height of the column, while the upper part with blue color indicates the polar component and the bottom part with orange color indicates the dispersive component of each crystal face. For the $( {11\bar{2}0} )$, $( {10\bar{1}0} )$ and $( {0001} )$ crystal faces, the polar component is around 47.3%, 42.9% and 31.7% of the total surface energy, indicating that these surfaces have an affinity to polar liquids; hence they are more hydrophilic. In comparison, the polar component of the $( {1\bar{1}02} )$ crystal face is <1% of the total surface energy, indicating that this crystal surface has more affinity to nonpolar liquids; hence it is more hydrophobic. On the other hand, Lewis acid and base portions were also analyzed with the van Oss-Good-Chaudhury method (Fig. 2d, Supplementary Table 3). The total surface energy and Lifshitz-van der Waals (LW) interaction for the $( {1\bar{1}02} )$ crystal face are slightly lower than those of the other three crystal faces. However, the Lewis base portion of the $( {1\bar{1}02} )$ crystal face decreased a lot, indicating that there are fewer electron donors on this crystal face compared with the other three. It was widely believed that the aluminum atom acts as a Lewis acid site because of the empty (3p) orbital on the valance band [29]. However, experimental results showed that the Lewis acid portions of all crystal faces are very small, possibly because of the adsorbed interfacial water molecules in ambient environments. Herein, it is intriguing to note that the $( {1\bar{1}02} )$ crystal face with the same chemical composition as $( {11\bar{2}0} )$, $( {10\bar{1}0} )$ and $( {0001} )$ crystal faces displays different macroscopic wettability properties with relatively low total surface energy, polar component and Lewis base portion.

**Figure 3.**
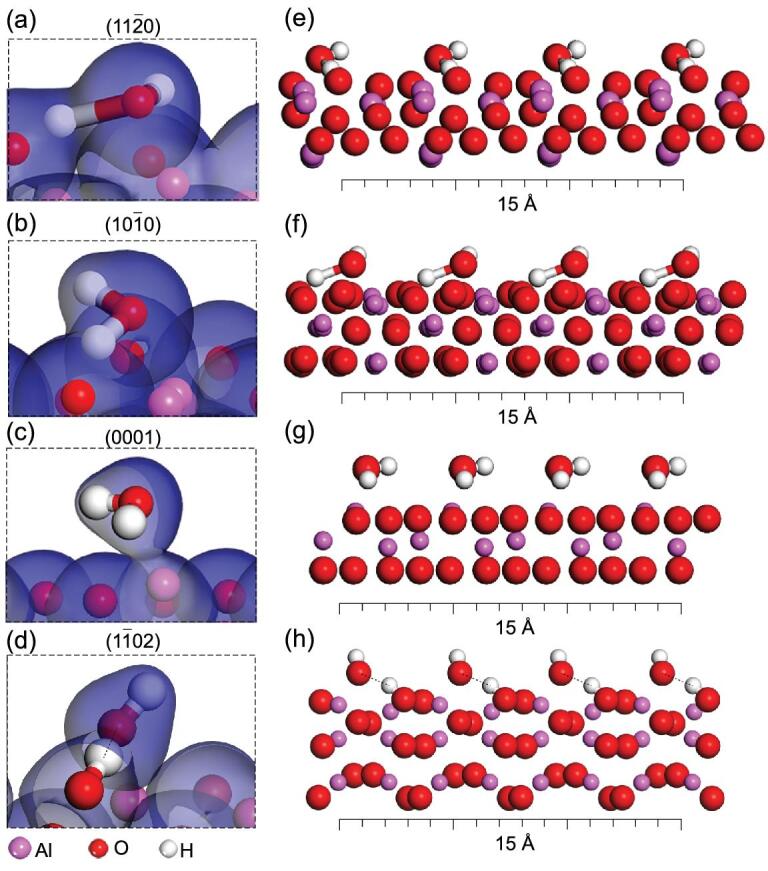
DFT simulation performed on the relaxed geometry of α-Al_2_O_3_ crystal faces. (a–d) Simulations of a single water molecule on α-Al_2_O_3_ crystal faces were performed, indicating that water molecules are at a dissociative state with one hydrogen atom at the top of the $( {1\bar{1}20} )$ crystal face, while oxygen atoms are at the top for the other three crystal faces. (e–h) Corresponding schematic diagrams of the first water layer film on each α-Al_2_O_3_ crystal surface were drawn according to the simulation results.

The wettability difference was further studied with DFT simulation at the molecular level. First principle calculations were performed on relaxed geometries of α-Al_2_O_3_ surfaces with one water molecule. Surface energies were calculated based on our model and compared with previous works, which verified the reliability of our results (Supplementary Fig. 3) [33–35]. As shown in Fig. 3a–d, when water molecules approach the $( {11\bar{2}0} )$, $( {10\bar{1}0} )$, $( {0001} )$ and $( {1\bar{1}20} )$ α-Al_2_O_3_ crystal faces, two typical water structures can be obtained for the final relaxed structures. Water molecules on the $( {11\bar{2}0} )$, $( {10\bar{1}0} )$ and $( {0001} )$ crystal faces interact strongly with the surface and keep a small distance from the exposed solid atoms. As shown in Fig. 3e–g, the oxygen atom is at the topmost position (also refer to the schematic diagram in Fig. 1b–d), which can offer two lone pair electrons to interact with the free water molecules from water droplets forming interfacial hydrogen-bond networks. Hence the three-phase contact line is eager to spread, and the solid surface is hydrophilic. While water molecules on the $( {1\bar{1}02} )$ crystal surface showed different behaviors compared with the other three (Fig. 3h). One hydrogen atom showed strong interaction with the top layer oxygen atom of the crystal surface, indicating that the adsorbed interfacial water molecule is in a dissociative state. At the same time, another hydrogen atom of the same water molecule is at the topmost position above the oxygen atom and the first hydrogen atom. There is only one hydroxyl group offering one interaction site for the free water molecules from water droplets to interact (also refer to the schematic diagram in Fig. 1e). In this case, the free water molecules from water droplets tend to form inner hydrogen bond networks rather than interacting with the adsorbed interfacial water molecules. The three-phase contact line is easily pinned, and the solid surface is relatively hydrophobic. Based on these simulation results, it can be seen that the adsorbed interfacial water molecules on different α-Al_2_O_3_ crystal faces typically possess two statuses. One is the hydrophilic state with an oxygen atom at the top to form interfacial hydrogen bond networks. In this case, the three-phase contact line will spread rather than pinning. The other is the hydrophobic state with one hydrogen atom at the top. In this situation, free water molecules from bulk water droplets at the three-phase contact line tend to form inner hydrogen-bond networks, and the three-phase contact line is eager to pin rather than spreading.

We also carried out DFT calculations for dual water molecules on the (0001) and $( {1\bar{1}02} ){\rm{\ }}$crystal faces of α-Al_2_O_3_. The results revealed that the orientation of the water as well as the charges of the system are critical components in interpretation of the CA at the macro scale. The water structure on the (0001) crystal face agrees well with the previous report (Supplementary Fig. 4) [36]. The structure is roughly hexagonal, with each of the H_2_O molecules located at a similar position above the Al atoms. Furthermore, Supplementary Fig. 5 shows the side and top view of the charges and water structures of the optimized two water molecules adsorbed on the $( {1\bar{1}02} ){\rm{\ }}$surface. As we can see, when two H_2_O molecules are added to the $( {1\bar{1}02} )$ surface, one of the H_2_O remains at a similar configuration and location to the case of single H_2_O which sits above and between Al and O. The H_2_O is distorted with OH attached to the Al and the other H close to O of the Al_2_O_3_ (Supplementary Fig. 5). Interestingly, the second H_2_O molecule is positioned above and between two Al atoms, and no distortion is observed. Various studies have already shown that charge is an important component in determination of the CA [37–39]. By accounting for the positions of the charges for different surfaces, differences in the surface dipole can be created and, as such, induce a difference in the dipole of the H_2_O molecules on top of the surface. The resulting dipole of the molecules, in turn, changes the way water interacts with itself (i.e. changing its shape). The difference in the CA between the (0001) and $( {1\bar{1}02} ){\rm{\ }}$directions can be explained by the difference in their total dipole of the dual H_2_O molecules in the out-of-plane direction, −0.776 vs. −0.007 Debye, indicating that the $( {1\bar{1}02} )\ $direction surface can only weakly interact with additional H_2_O molecules (Supplementary Figs 6 and 7).

**Figure 4.**
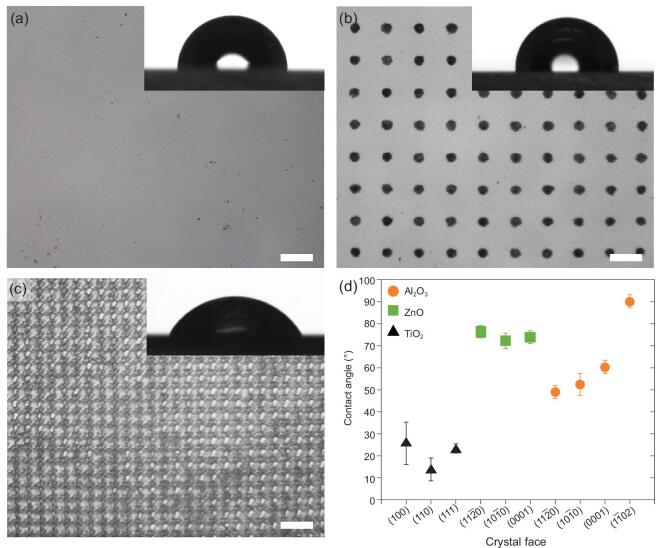
Optical images of laser engraving patterned $( {1\bar{1}02} )$ crystal faces (scale bar is 100 μm) and further CA measurements of other metal oxide surfaces. (a) Plane α-Al_2_O_3_$( {1\bar{1}02} )$ crystal face with water CAs of 90.5 ± 2.4°. (b) Laser engraving patterns of 20 μm dots with 100 μm inter-pore distance and water CAs of 96.2 ± 2.0°. (c) Laser engraving patterns of 20 μm dots with no gap and water CAs of 62.5 ± 2.9°. (d) CA measurements of crystal face dependent wettability difference for TiO_2_, ZnO and α-Al_2_O_3_.

The impact of surface roughness and the hydrophilic crystal faces were evaluated by generating laser engraved hole patterns on α-Al_2_O_3_$( {1\bar{1}02} )$ crystal faces. As shown in Fig. 4a–c, the diameter of the laser mark dot is around 20 μm, while the inter-pore distances are 100 μm and 0 μm, respectively. Water CA measurements were applied (insets in Fig. 4a–c). It is interesting to note that when the inter-pore distance is decreased, the water CAs will increase at first from 90.5 ± 2.4° to 96.2 ± 2.0° and then decrease from 96.2 ± 2.0° to 62.5 ± 2.9°. It is assumed that this intriguing phenomenon may result from competition between surface roughness and the exposure of hydrophilic crystal faces. When the inter-pore distance is 100 μm, an air cushion may form below the water droplet resulting in a Cassie-Baxter state. Comparatively, when the inter-pore distance is 0 μm, the air cushion can be removed, and the wetting state changes from Cassie-Baxter state to Wenzel state. In this case, the wettability contribution of hydrophilic crystal faces becomes dominant for the macroscopic wettability rather than the air cushion induced by surface roughness. Besides, a similar crystal face dependent wettability difference can also be found on other metal oxides such as TiO_2_ and ZnO (Fig. 4d). Based on this principle, it is inferred that the wettability of crystal surfaces would be defined more precisely with crystal face and environmental conditions concerning the orientation of adsorbed interfacial water molecules as long as the exposed solid atoms at the surfaces.

In summary, crystal face dependent intrinsic wettability of metal oxide surfaces was investigated, typically $( {11\bar{2}0} )$, $( {10\bar{1}0} )$, $( {0001} )$ and $( {1\bar{1}02} )$ crystal faces of α-Al_2_O_3_. It is intriguing to find that, for these atomic flat surfaces with similar chemical compositions, water CAs of these crystal faces change from hydrophilic to hydrophobic. The unique wettability difference was analyzed concerning surface energy with various methods, and it was found that the $( {1\bar{1}02} )$ crystal face has the lowest total surface energy, polar component and Lewis base portion compared with the other three. Furthermore, DFT simulation reveals that the wettability difference can be explicated concerning orientations of adsorbed interfacial water molecules, which determines whether the three-phase contact line tends to spread or pin from the perspective of molecular level. This work demonstrated that the adsorbed interfacial molecules have a significant role in macroscopic wettability, which can serve as another critical factor for atomic flat surfaces. The properties of solid surfaces (atoms structures, surface unsaturation electrons, functional groups, polar and dispersive components, Lewis acid/base portions, etc.) will firstly affect the orientation of adsorbed interfacial molecules and then determine the macroscopic wettability.

## METHODS

### Materials and cleaning process

α-Al_2_O_3_ crystal faces with $( {11\bar{2}0} )$, $( {10\bar{1}0} )$, $( {0001} )$ and $( {1\bar{1}02} )$ orientations were purchased from Shanghai Institute of Optics and Fine Mechanics, CAS. They had an inside length of 10 mm and were 0.5 mm thick, polished on one side with <5 Å roughness on average. The water used was of high purity with resistivity of 18.2 MΩ cm and surface tension of around 72.6 mN/m at 22°C. Acetone was purchased from Beijing Shiji. Formamide was purchased from Sigma-Aldrich. Diiodomethane was purchased from Alfa Aesar. All reagents were used as received without further purification.

α-Al_2_O_3_ crystals were polished again on silk surface with acetone, rinsed with acetone, sonicated in deionized water, and then dried in a stream of high purity N_2_. CA measurements were carried out immediately after the cleaning procedure.

### Surface characterizations

CAs for the three probe liquids of deionized water, formamide, and diiodomethane were measured using an SCA 25 machine (Data-Physics, Germany) at ambient temperature. CAs were taken as an average of three measurements on different positions of crystal surfaces. Drops of 2 μL at room temperature were deposited by a micro-syringe pointed vertically down onto the sample surface. The surface energies of Al_2_O_3_ single crystals were calculated using the Equation-of-State method, Owens-Wandt-Rabel-Kaelble method, van Oss-Good-Chaudhury method and Wu's harmonic Mean method. All single crystal samples were characterized by X-ray diffraction (GBC-MMA) over 2θ angles in the range 10–90° using CuKα radiation (1.54059 Å).

### Computational method

In this work, first-principle calculations were performed using density functional theory (DFT) implemented using the CASTEP package [33–35]. The exchange-correlation function used to describe the exchange-correlation interaction was the General Gradient Approximation (GGA) with the Perdew–Burke–Ernzerhof (PBE) formulation [40], a van der Waals (vdW) correction (DFT-D) implemented by Grimme was added [41]. Structures were optimized using the Broyden–Fletcher–Goldfarb–Shanno algorithm BFGS [42].

### Surface morphology fabrication

Surface morphologies were fabricated using a UV laser engraving machine (Han's Laser Technology Co., Ltd) with a wavelength of 355 nm. The laser beam was finally focused by a lens. The maximum mean power of the laser system was 5 W in a Gaussian beam mode with a beam quality factor M^2^ ≤ 1.3. The opto-acoustical Q-switch commutator controlled the cavity output in continuous and in pulsed mode, generating a pulse range of 80–260 ns with a frequency range of 1–40 kHz. The machining process was controlled by the diode pump current intensity (in relation to peak power), pulse frequency, scanning speed and fill spacing. The relationship between the laser power and the current intensity was determined by measuring the power at different levels of current intensity. The values of the average power were measured at different levels of current density when pulse frequency was 1, 3 and 5 kHz, respectively. The scanning speed was 40 mm/s.

## Supplementary Material

nwaa166_Supplemental_File

## Notes

***Con* *fl* *ict of* *interest statement*.** None declared.
