# Supplementary material for: Crystal face dependent intrinsic wettability of metal oxide surfaces
Source: Natl Sci Rev. 2020 Jul 18;8(1):nwaa166. doi: 10.1093/nsr/nwaa166 (PMC8288373; doi:10.1093/nsr/nwaa166)
Supplement: nwaa166_Supplemental_File [file nwaa166_supplemental_file.docx]

**Supplementary Information**

**Crystal Face Dependent Intrinsic Wettability of Metal Oxide Surfaces**

Zhongpeng Zhu^1,3^, Zhenwei Yu^2,3^, Frank F. Yun^2^, Deng Pan^5^, Ye Tian^*,1,4^, Lei Jiang,^1,3,4^ and Xiaolin Wang ^*,2^

^1^Key Laboratory of Bio-inspired Materials and Interfacial Science, Technical Institute of Physics and Chemistry, Chinese Academy of Sciences, Beijing 100190, China;

^2^Institute for Superconducting and Electronic Materials, Australian Institute for Innovative Materials, University of Wollongong, Wollongong, NSW 2500, Australia;

^3^Key Laboratory of Bio-inspired Smart Interfacial Science and Technology of Ministry of Education, School of Chemistry, Beihang University, Beijing 100191, China;

^4^University of Chinese Academy of Sciences, 100049 Beijing, China;

^5^Shandong Yi An Bio-Engineering Co., LTD.

*Corresponding author:

tianyely@iccas.ac.cn; xiaolin@uow.edu.au.

**Supplementary Figures**


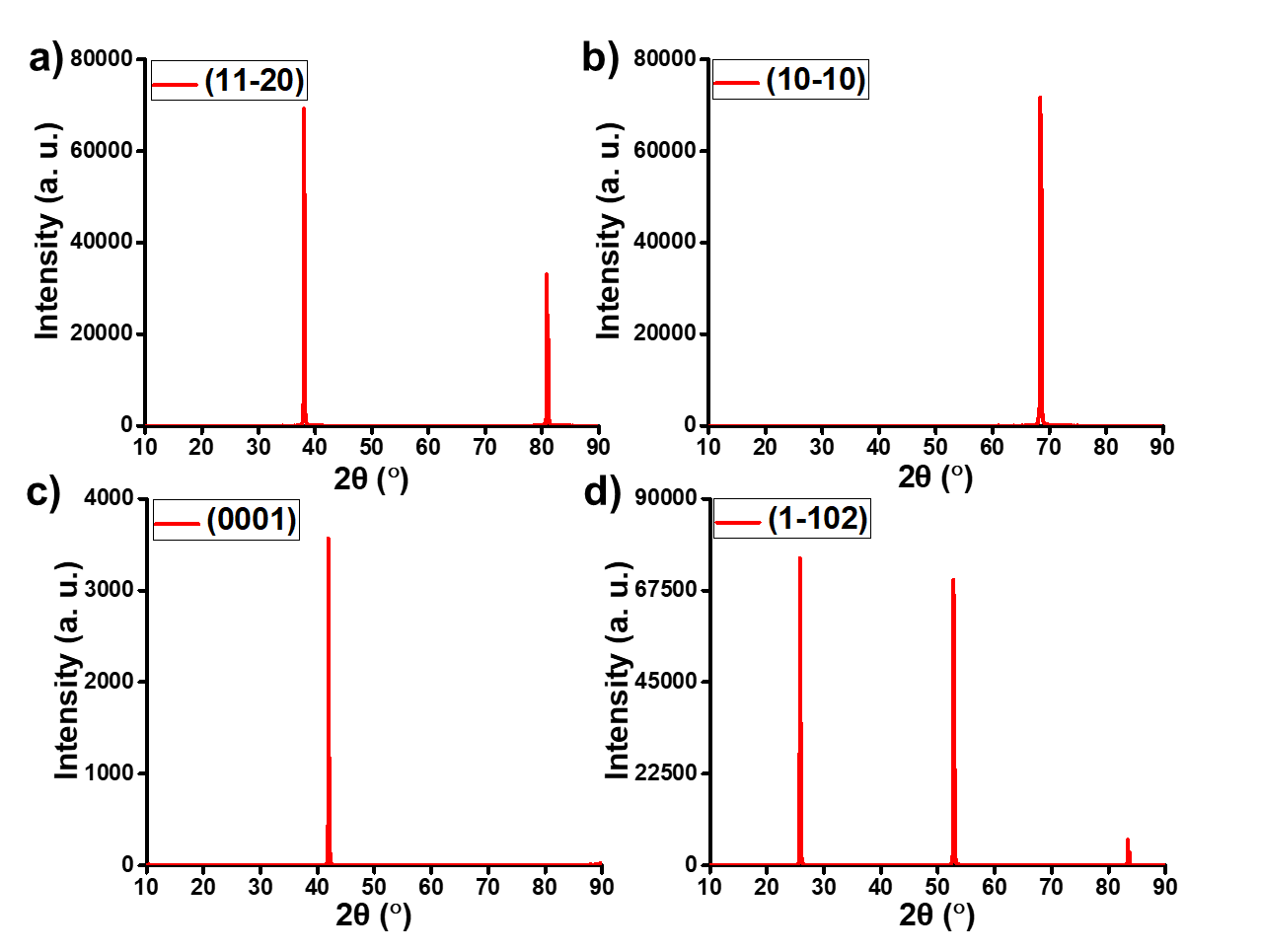


**Supplementary Figure 1.** X-ray diffraction patterns of α-Al_2_O_3_ single crystal surfaces with (a) $(11\bar{2}0)$, (b) $(10\bar{1}0)$, (c) $(0001)$ and (d) $(1\bar{1}02)$ orientations.


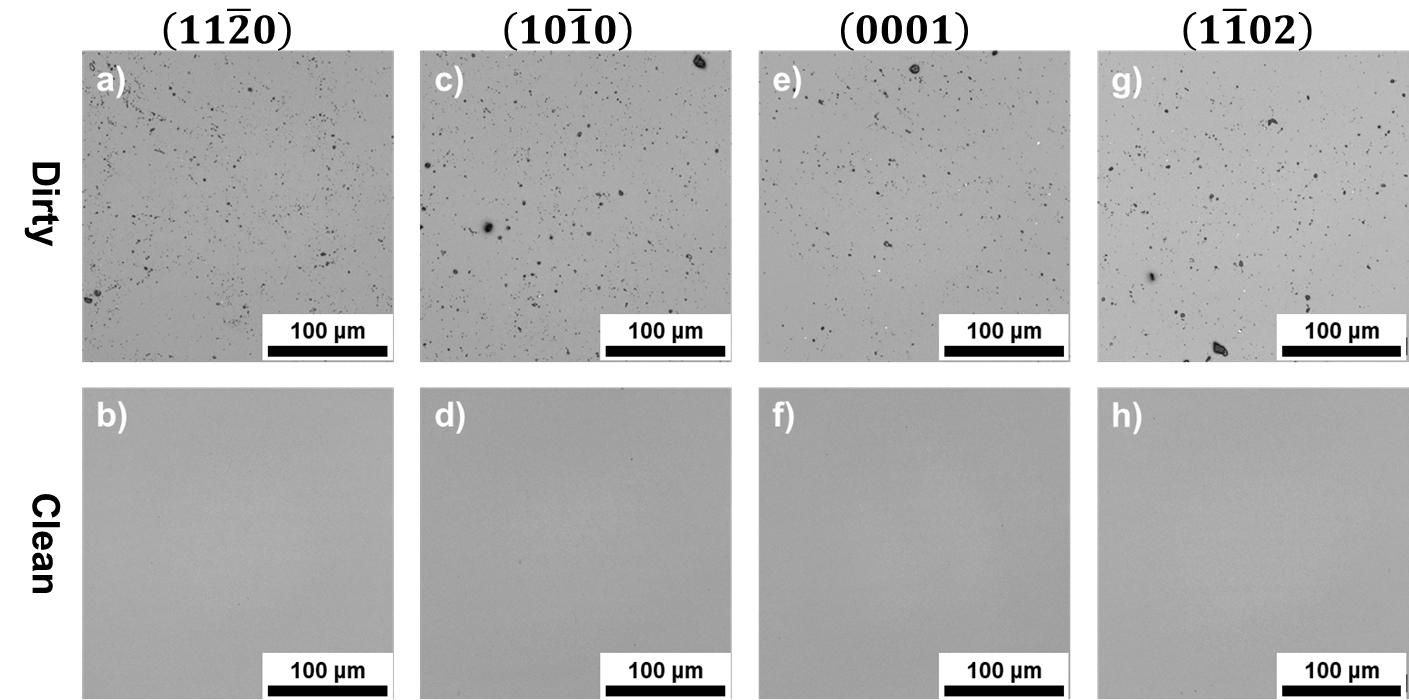


**Supplementary Figure 2.** Optical images of original and treated α-Al_2_O_3_ single crystal surfaces which indicates that the crystal surfaces are all clean.


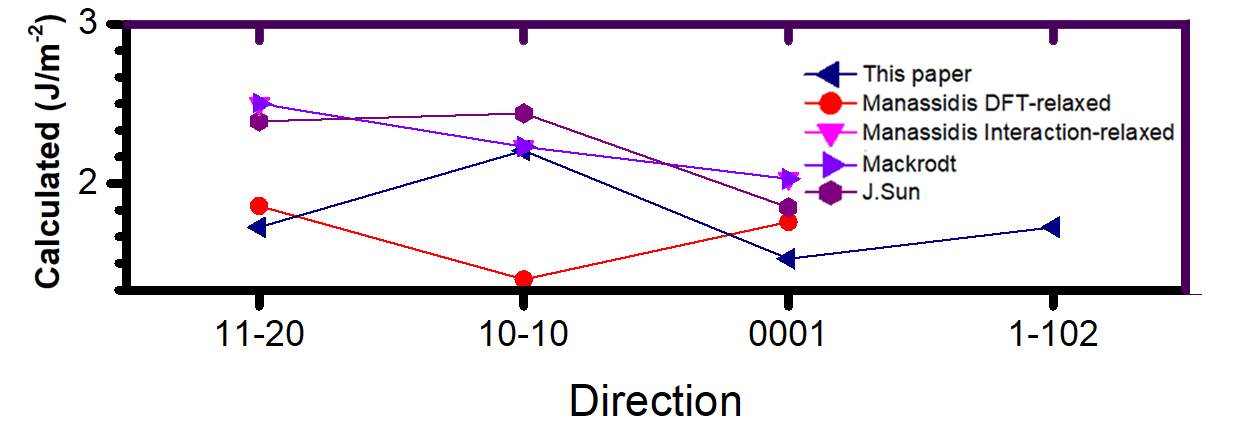


**Supplementary Figure 3.** Surface energies are calculated based on our model and compared with the reported results. The surface energies of all α-Al_2_O_3_ crystal surfaces have lain within a range between 1-3 J m^-2^. It can be seen that the trend of the data matches well with the data of J. Sun, while the range matches well with the data of Manassidis. [[1-3](#_ENREF_1)]


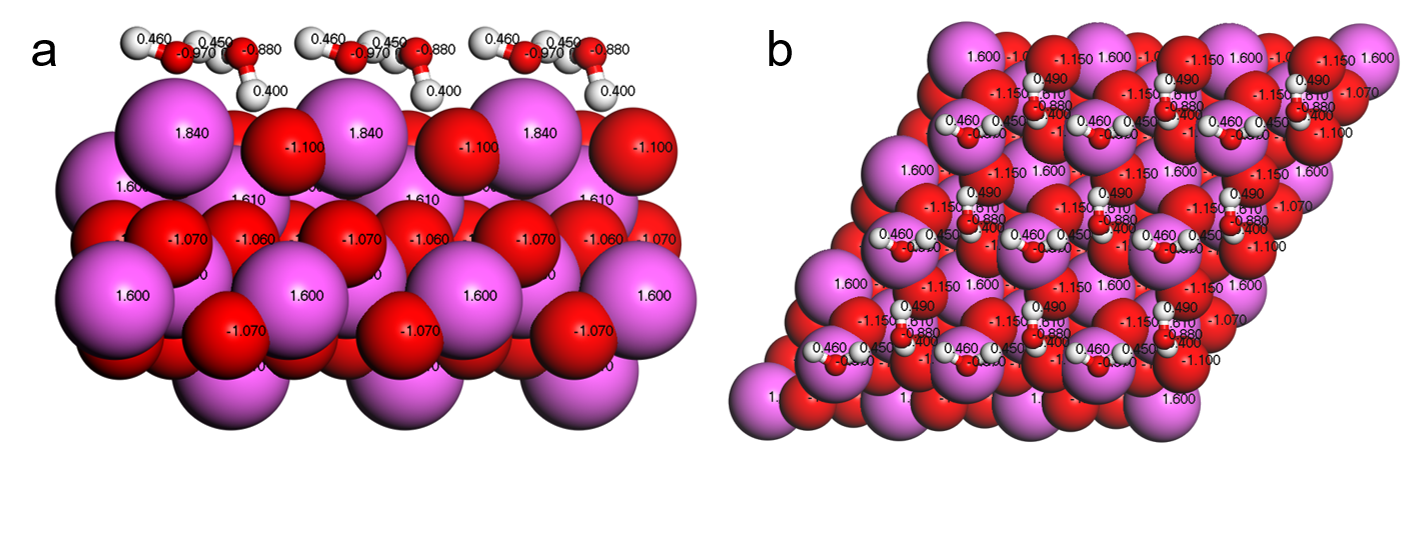


**Supplementary Figure 4.** Side (c) and top (d) views of the charges and H_2_O structure of the optimized dual water molecule adsorption on the (0001) surface.


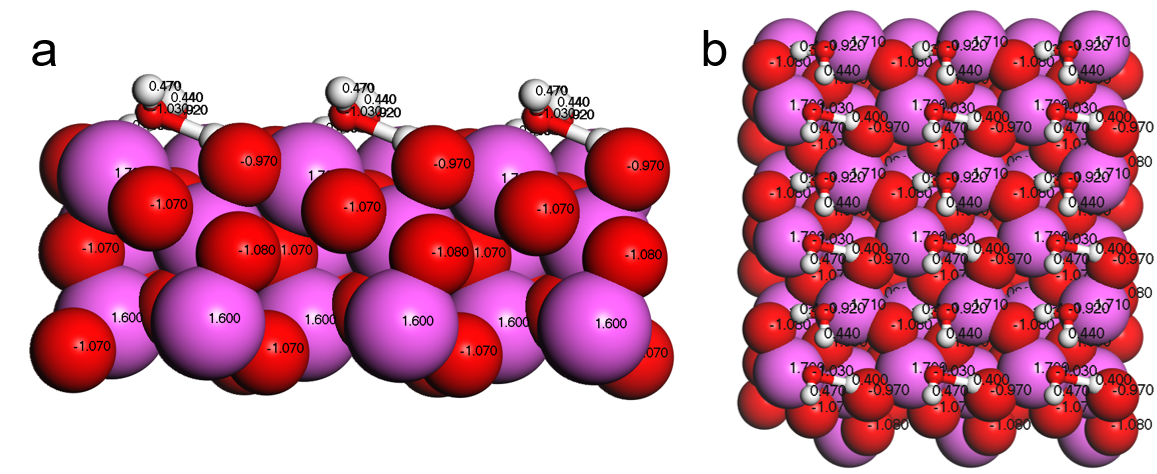


**Supplementary Figure 5.** Side (c) and top (d) views of the charges and H_2_O structure of the optimized dual water molecule adsorption on the $\left( 1\bar{1}02 \right)$ surface.


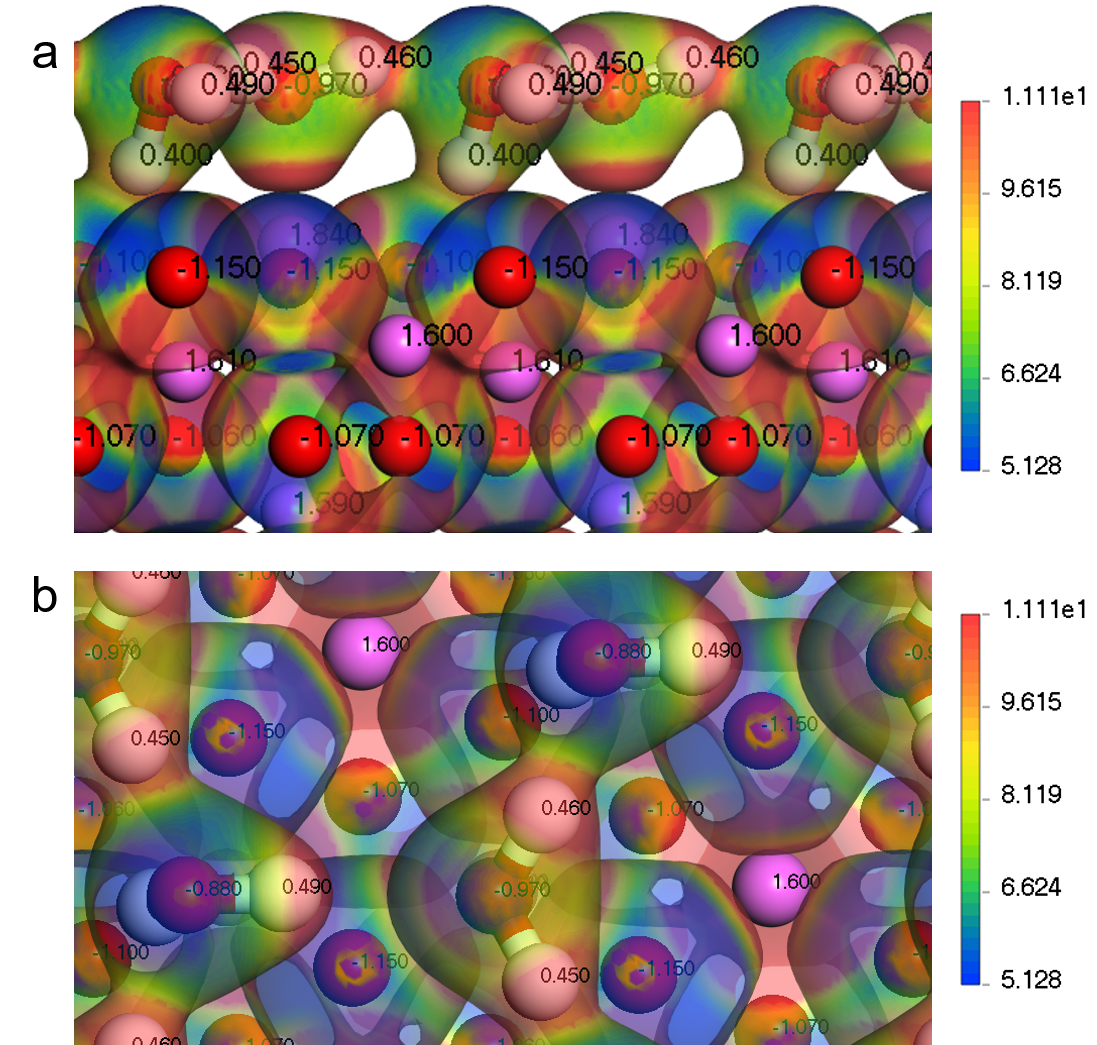


**Supplementary Figure 6.** Side (a) and top (b) views of the electron density and mapped potentials of dual water molecule adsorption on the (0001) surface.


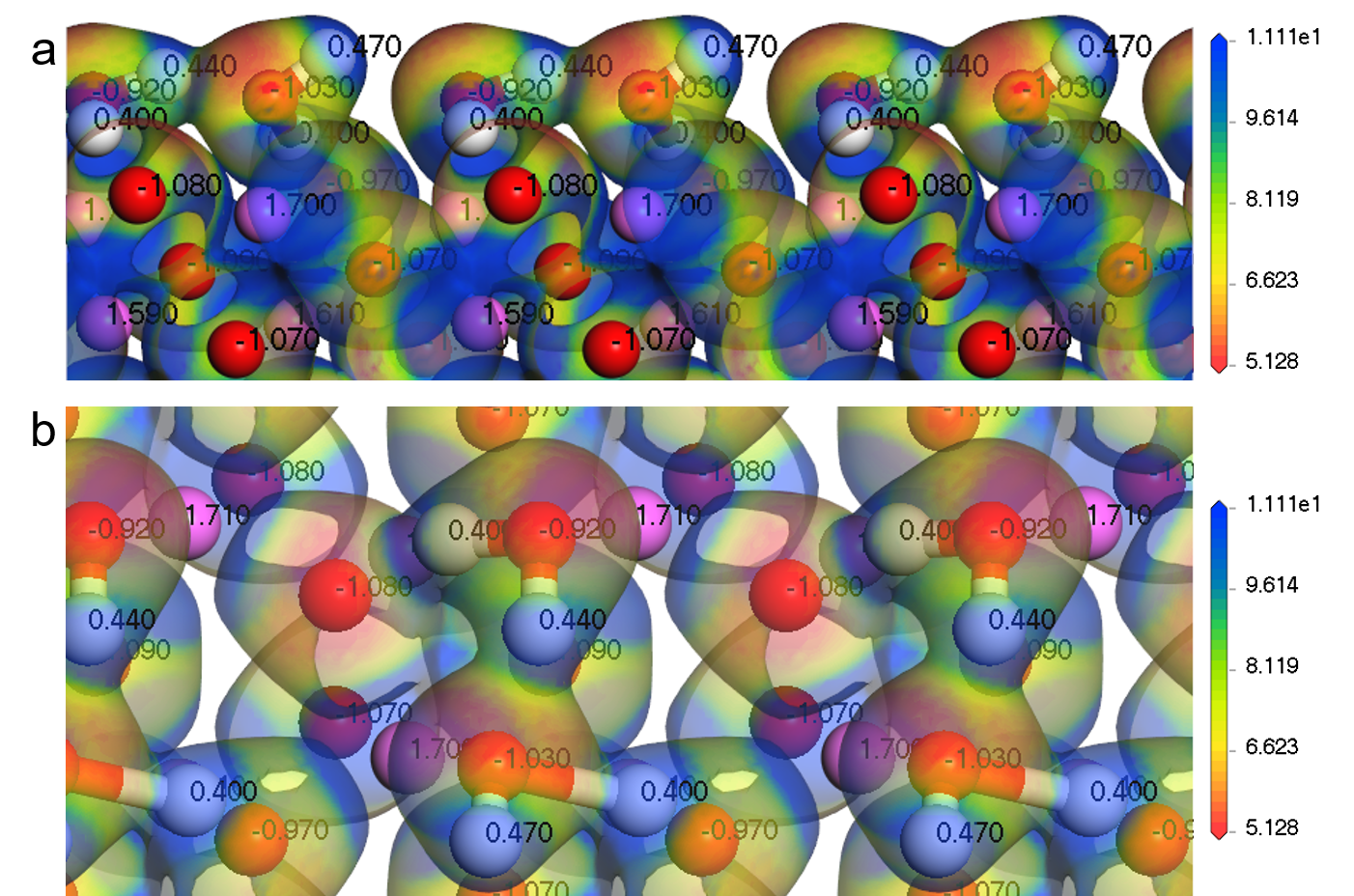


**Supplementary Figure 7.** Side (a) and top (b) views of the electron density and mapped potentials of dual water molecule adsorption on the $\left( 1\bar{1}02 \right)$ surface.

**Supplementary Table 1.** Measured contact angles of three liquids on Al_2_O_3_ single crystals and the surface energies of Al_2_O_3_ single crystals are calculated using the Equation-of-State method.

| **Surfaces** | **Measured contact angles (°)** | | | **Surface Energy(mN/m)** |
| --- | --- | --- | --- | --- |
|  | **Water** | **Formamide** | **Diiodomethane** |  |
| Al_2_O_3_ $(11\bar{2}0)$ | 50.0±3.1 | 50.1±3 | 40.3±2 | 46.35 |
| Al_2_O_3_ $(10\bar{1}0)$ | 54.3±4.7 | 50.3±3 | 40.4±3 | 45.43 |
| Al_2_O_3_ $(0001)$ | 60.4±3.0 | 51.4±2 | 40.9±1 | 43.23 |
| Al_2_O_3_ $(1\bar{1}02)$ | 90.2±2.5 | 68.1±5 | 46.9±2 | 33.43 |

**Supplementary Table 2.** The surface energies of Al_2_O_3_ single crystals are calculated using the Owens-Wandt-Rabel-Kaelble (OWRK) method.

| **Surfaces** | **Surface Energy and its components (mN/m)** | | |
| --- | --- | --- | --- |
|  | **Surface Energy** | **Dispersive** | **Polar** |
| Al_2_O_3_ $(11\bar{2}0)$ | 47.84 | 25.23 | 22.61 |
| Al_2_O_3_ $(10\bar{1}0)$ | 46.12 | 26.36 | 19.77 |
| Al_2_O_3_ $(0001)$ | 42.56 | 29.06 | 13.51 |
| Al_2_O_3_ $(1\bar{1}02)$ | 36.25 | 35.91 | 0.34 |

**Supplementary Table 3.** The surface energies of Al_2_O_3_ single crystals are calculated using the van Oss-Good-Chaudhury method.

| **Surfaces** | **Surface Energy and its components (mN/m)** | | | |
| --- | --- | --- | --- | --- |
|  | **Surface Energy** | **LW** | **Acid** | **Base** |
| Al_2_O_3_ $(11\bar{2}0)$ | 38.78 | 38.78 | 0 | 37.77 |
| Al_2_O_3_ $(10\bar{1}0)$ | 39.07 | 39.07 | 0 | 33.76 |
| Al_2_O_3_ $(0001)$ | 40.16 | 39.15 | 0.01 | 23.82 |
| Al_2_O_3_ $(1\bar{1}02)$ | 35.9 | 35.9 | 0 | 2.69 |

**Supplementary Table 4.** The surface energies of Al_2_O_3_ single crystals are calculated using the Wu’s harmonic Mean method.

| **Surfaces** | **Surface Energy and its components (mN/m)** | | |
| --- | --- | --- | --- |
|  | **Surface Energy** | **Dispersive** | **Polar** |
| Al_2_O_3_ $(11\bar{2}0)$ | 50.62 | 30.42 | 20.2 |
| Al_2_O_3_ $(10\bar{1}0)$ | 49.27 | 30.88 | 18.39 |
| Al_2_O_3_ $(0001)$ | 46.25 | 31.92 | 14.33 |
| Al_2_O_3_ $(1\bar{1}02)$ | 36.59 | 34.4 | 2.19 |

**References**

1. Sun J, Stirner T, Matthews A. Structure and surface energy of low-index surfaces of stoichiometric α-Al_2_O_3_ and α-Cr_2_O_3_. *Surf Coat Technol*. 2006; **201**: 4205-8.

2. Manassidis I, Gillan MJ. Structure and Energetics of Alumina Surfaces Calculated from First Principles. *J Am Ceram Soc*. 1994; **77**: 335-8.

3. Mackrodt WC, Davey RJ, Black SN*, et al.* The morphology of α-Al_2_O_3_ and α-Fe_2_O_3_: The importance of surface relaxation. *J Cryst Growth*. 1987; **80**: 441-6.

4. Thissen P, Grundmeier G, Wippermann S*, et al.* Water adsorption on the alpha-Al_2_O_3_(0001) surface. *Phys Rev B*. 2009; **80**: 6.
